# Small-molecule PS10 inhibits PRRSV replication by targeting HSP90 and multiple viral non-structural proteins

**DOI:** 10.1128/jvi.02121-25

**Published:** 2026-02-18

**Authors:** Yongjie Chen, Jingxing Wang, Haotong Lu, Zishen Chen, Baoying Huang, Chunhe Guo

**Affiliations:** 1 Guangdong Laboratory for Lingnan Modern Agriculture, State Key Laboratory of Animal Disease Control and Prevention, Key Laboratory of Zoonosis Prevention and Control of Guangdong Province, College of Veterinary Medicine, South China Agricultural University 12526, , Guangzhou, Guangdong, People's Republic of China; University of Kentucky College of Medicine, Lexington, Kentucky, USA

**Keywords:** PRRSV, PS10, HSP90, viral non-structural proteins

## Abstract

Livestock production is vital to global food security but faces major threats from pathogens such as porcine reproductive and respiratory syndrome virus (PRRSV), which causes substantial economic losses. The genetic diversity of PRRSV and limited cross-protection among strains complicate vaccination strategies, highlighting the urgent need for effective antiviral treatments. In this study, we screened a compound library and identified 2-[(2,4-dihydroxyphenyl) sulfonyl] isoindoline-4,6-diol (designated PS10) as a potent inhibitor of PRRSV replication. PS10 exhibited dose-dependent antiviral effects in both Marc-145 cells and primary porcine alveolar macrophages, with efficacy independent of the viral strain. Further mechanistic studies revealed that PS10 acts specifically during the replication phase of the viral life cycle. Notably, PS10 significantly suppressed PRRSV infection-induced heat shock protein 90 (HSP90) expression, a critical host factor for viral replication. This suppression not only impaired viral propagation but also attenuated the production of pro-inflammatory cytokines. Furthermore, PS10 selectively reduced the abundance of multiple viral non-structural proteins (nsp2, nsp3, nsp10, and nsp11). The binding of PS10 to these proteins was confirmed by molecular docking and cellular thermal shift assays, which underlie the observed inhibition. This study not only expands our understanding of PS10’s antiviral mechanisms but also provides new insights into host factor regulation of viral replication, offering potential avenues for developing broad-spectrum antiviral therapies.

**IMPORTANCE:**

Porcine reproductive and respiratory syndrome virus (PRRSV) causes substantial economic losses to the global swine industry. Current vaccines often lack cross-protection against heterologous strains, underscoring the need for broad-spectrum alternatives. We identified PS10 through compound screening as a potent inhibitor of PRRSV replication with low cytotoxicity. Mechanistically, PS10 significantly suppressed virus-induced expression of heat shock protein 90 (HSP90). This suppression not only inhibited viral propagation but also reduced pro-inflammatory cytokine production. Furthermore, PS10 reduced the levels of multiple viral non-structural proteins (nsp2, nsp3, nsp10, and nsp11). The binding of PS10 to these proteins was confirmed by molecular docking and cellular thermal shift assays. Taken together, our results suggest that PS10 is a promising candidate for controlling PRRSV infection and may provide a foundation for developing broad-spectrum antiviral agents.

## INTRODUCTION

As a primary source of meat in China, pork plays a vital role in the national diet and agricultural economy. However, the emergence and re-emergence of infectious diseases have posed serious challenges to the global swine industry. Viral diseases, in particular, threaten the sustainable development of pig farming and food security due to their high contagiousness and potential for widespread transmission (1, 2). Among these, porcine reproductive and respiratory syndrome (PRRS), caused by the PRRS virus (PRRSV), is one of the most significant viral threats (3, 4). First identified in the United States in 1987, PRRS has spread worldwide, severely constraining pig production and causing substantial economic losses (5–7). PRRSV is an enveloped virus containing a single-stranded, positive-sense RNA genome approximately 15 kb in length. The genome comprises at least 11 open reading frames that encode 12 non-structural proteins (nsps) essential for viral replication, along with eight structural proteins required for virion assembly (8). Although pigs of all ages are susceptible to PRRSV infection, the most severe clinical outcomes are observed in pregnant sows and neonatal piglets (9). The virus exhibits specific tropism for macrophages, particularly porcine alveolar macrophages (PAMs), where it replicates before disseminating to lymphatic tissues and the lungs, thereby inducing respiratory symptoms. *In vitro*, it is capable of infecting the African green monkey kidney cell line (MA-104) and its derivative cell lines, such as Marc-145 (10).

PRRSV, as an RNA virus, exhibits characteristically high mutation rates due to the absence of proofreading mechanisms in its RNA-dependent RNA polymerase (11). This enables the virus to evolve rapidly, creating numerous genetic variants and resulting in extensive genetic and antigenic diversity among strains and leading to poor cross-protection between heterologous strains. Coupled with its immune evasion ability, current vaccines often fail to provide broad and lasting protection (12, 13). Given the limitations of these vaccines, small-molecule antiviral compounds are gaining attention as potential therapeutic alternatives for PRRSV control. These compounds can be specifically designed to target critical viral proteins or host factors. For example, small-molecule inhibitors can bind to the viral protease, blocking the processing of the viral polyprotein and inhibiting replication (14–16). Alternatively, other inhibitors may target essential host proteins involved in viral replication pathways (17, 18). Importantly, as long as target sites remain conserved across strains, these compounds can overcome viral variability. Therefore, screening for such compounds offers a promising strategy against PRRSV. Our experimental data demonstrate that the small-molecule 2-[(2,4-dihydroxyphenyl) sulfonyl] isoindoline-4,6-diol (PS10) significantly inhibits PRRSV replication, although its antiviral mechanism remains to be elucidated. Interestingly, PS10 has been previously identified as a novel ATP-competitive broad-spectrum pyruvate dehydrogenase kinase (PDK) inhibitor with demonstrated therapeutic potential for diabetic cardiomyopathy (19). Recent studies have also identified heat shock protein 90 (HSP90) as a binding target of PS10 (20). However, the function of PS10 in viral infection processes, particularly its antiviral mechanisms against PRRSV, remains unexplored. Further exploration of PS10’s inhibitory effects on PRRSV replication may not only reveal novel antiviral mechanisms but also provide valuable insights for developing effective PRRSV control strategies.

In our research, through compound library screening, we identified PS10 as a potent inhibitor of PRRSV infection. Our mechanistic investigation revealed that PS10 inhibits PRRSV-induced HSP90 expression, which interferes with viral replication and reduces pro-inflammatory cytokine production. Additionally, PS10 was shown to selectively target and decrease the protein abundance of several viral nsps (nsp2, nsp3, nsp10, and nsp11). Furthermore, the interaction between PS10 and these nsps may contribute to their inhibition. These results highlight the potential of targeting both host HSP90 and viral nsps as a novel antiviral approach against PRRSV.

## RESULTS

### PS10 significantly inhibits PRRSV infection in Marc-145 cells

**Fig 1:**
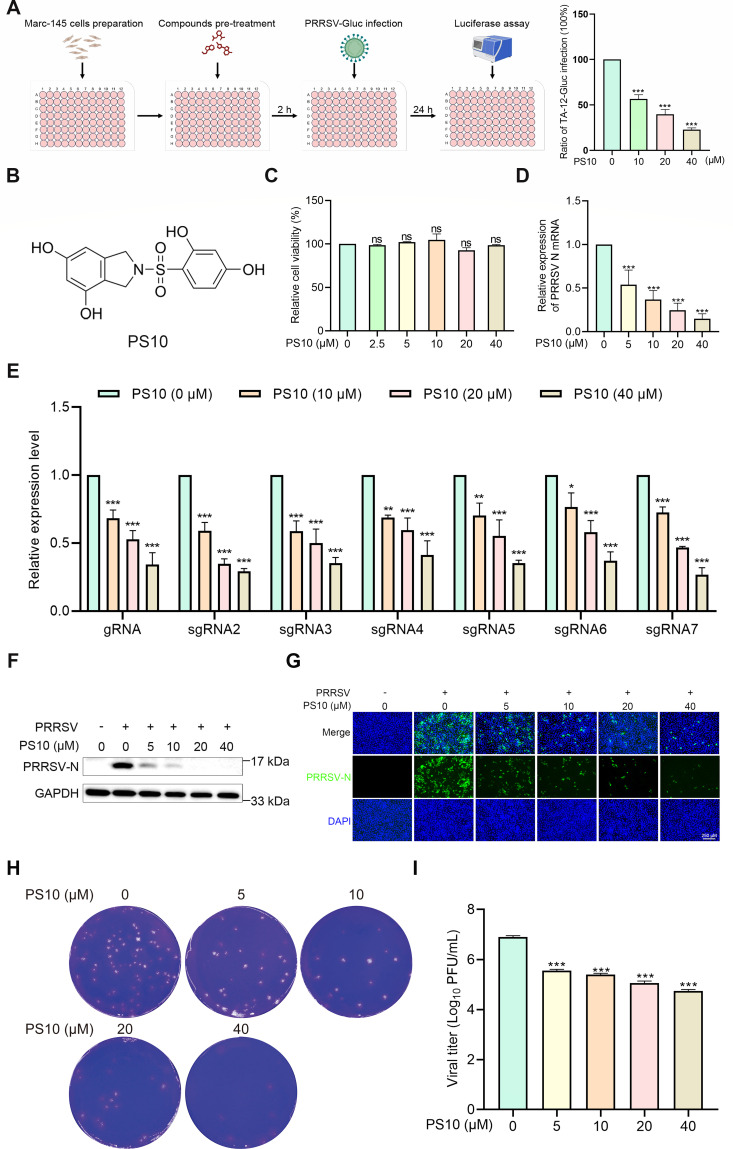
PS10 inhibits PRRSV infection in Marc-145 cells in a dose-dependent manner. (**A**) Design schematic of the compound screening experiment against PRRSV infection. Marc-145 cells were pre-incubated with the compound for 2 h and then infected with rTA-Gluc2 for 24 h. Culture supernatants were collected, and viral load was quantified using a microplate reader. (**B**) Chemical structure of PS10. (**C**) After 24 h of treatment with PS10 at the indicated concentrations, the cytotoxicity of PS10 on Marc-145 cells was assessed using a CCK-8 assay. (**D-I**) Marc-145 cells were treated with PS10 for 2 h and then infected with PRRSV CH-1a (MOI = 0.5). After 24 h, cells and supernatants were harvested. Viral RNA levels were measured by RT-qPCR (**D and E**). Viral N protein expression was analyzed by Western blotting (**F**) or immunofluorescence assay (IFA) (**G**). Virus titers in the supernatants were determined by plaque assay on Marc-145 cells (**H and I**). ***P < 0.001; **P < 0.01; *P < 0.05; ns: not significant.

**Fig 2:**
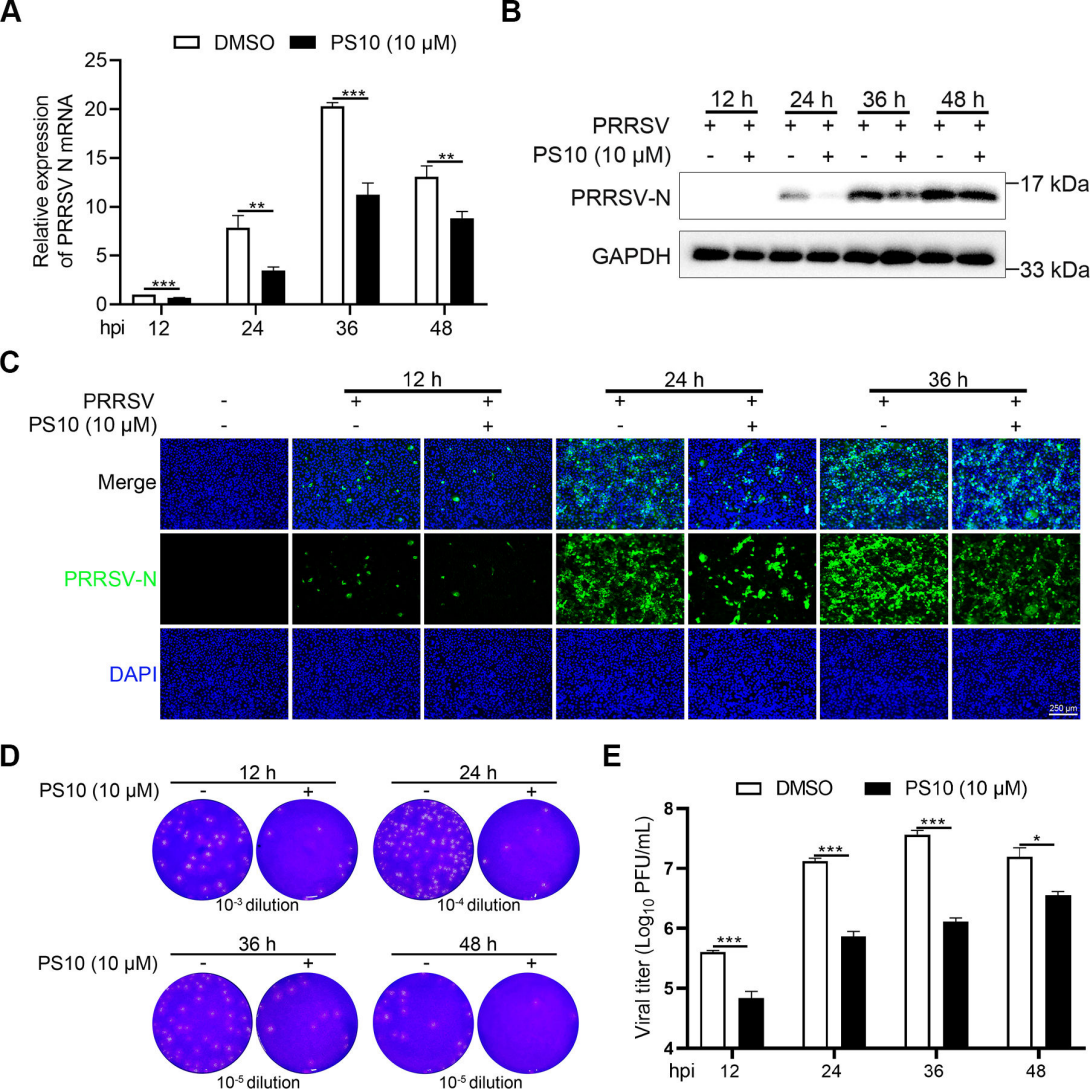
Time-dependent inhibition of PRRSV replication by PS10 in Marc-145 cells. (**A–E**) Marc-145 cells were treated with PS10 (10 μM) for 2 h and then infected with PRRSV CH-1a (MOI = 0.5) at the indicated time points, after which cells and supernatants were harvested for analysis. Viral RNA levels were measured by RT-qPCR (**A**). Viral N protein expression was analyzed by Western blotting (**B**) or IFA (**C**). Virus titers in the supernatants were determined by plaque assay on Marc-145 cells (**D and E**). ***P < 0.001; **P < 0.01; *P < 0.05; ns: not significant.

Small-molecule compound screening has been widely employed to identify inhibitors and therapeutic candidates for the prevention and treatment of diverse viral infections. To identify PRRSV inhibitors, Marc-145 cells were pretreated with compounds (10 μM) for 2 h before infection with luciferase-expressing PRRSV at a multiplicity of infection (MOI) of 0.5. Measurement of Gluc activity at 24 h post-infection (hpi) showed that PS10 significantly reduced Gluc expression, indicating strong anti-PRRSV activity (Fig. 1A). To exclude potential cytotoxic effects on viral infection, we first evaluated the impact of PS10 on Marc-145 cell viability using a CCK-8 assay (Fig. 1B). No significant cytotoxicity was observed at PS10 concentrations ≤ 40 µM (Fig. 1C). We next examined the antiviral activity of PS10 against the PRRSV CH-1a strain. Although PS10 inhibited the mRNA levels of PRRSV N in a dose-dependent manner (Fig. 1D), the primers used targeted the N gene sequence and thus could not distinguish between genomic RNA (gRNA) and nested subgenomic mRNAs (sgmRNAs). To dissect its specific effect, we employed targeted assays for viral RNA synthesis and found that PS10 significantly suppressed the levels of both gRNA and sgmRNA (Fig. 1E). These results demonstrate that PS10 inhibits not only viral genomic replication but also subgenomic transcription. PS10 also reduced the level of the PRRSV N protein (Fig. 1F and G). Correspondingly, viral titers were significantly reduced in a dose-dependent manner compared to the control group (Fig. 1H and I). We further investigated the antiviral efficacy of PS10 at different time points during PRRSV infection. The results demonstrated that PS10 consistently suppressed both mRNA and protein levels of the PRRSV N across all tested time points (Fig. 2A through C). Furthermore, plaque assays confirmed that PS10 significantly reduced viral titers at each infection time point examined (Fig. 2D and E). Collectively, these data demonstrate that PS10 significantly inhibits PRRSV replication in Marc-145 cells.

### PS10 suppresses PRRSV infection in PAMs

**Fig 3:**
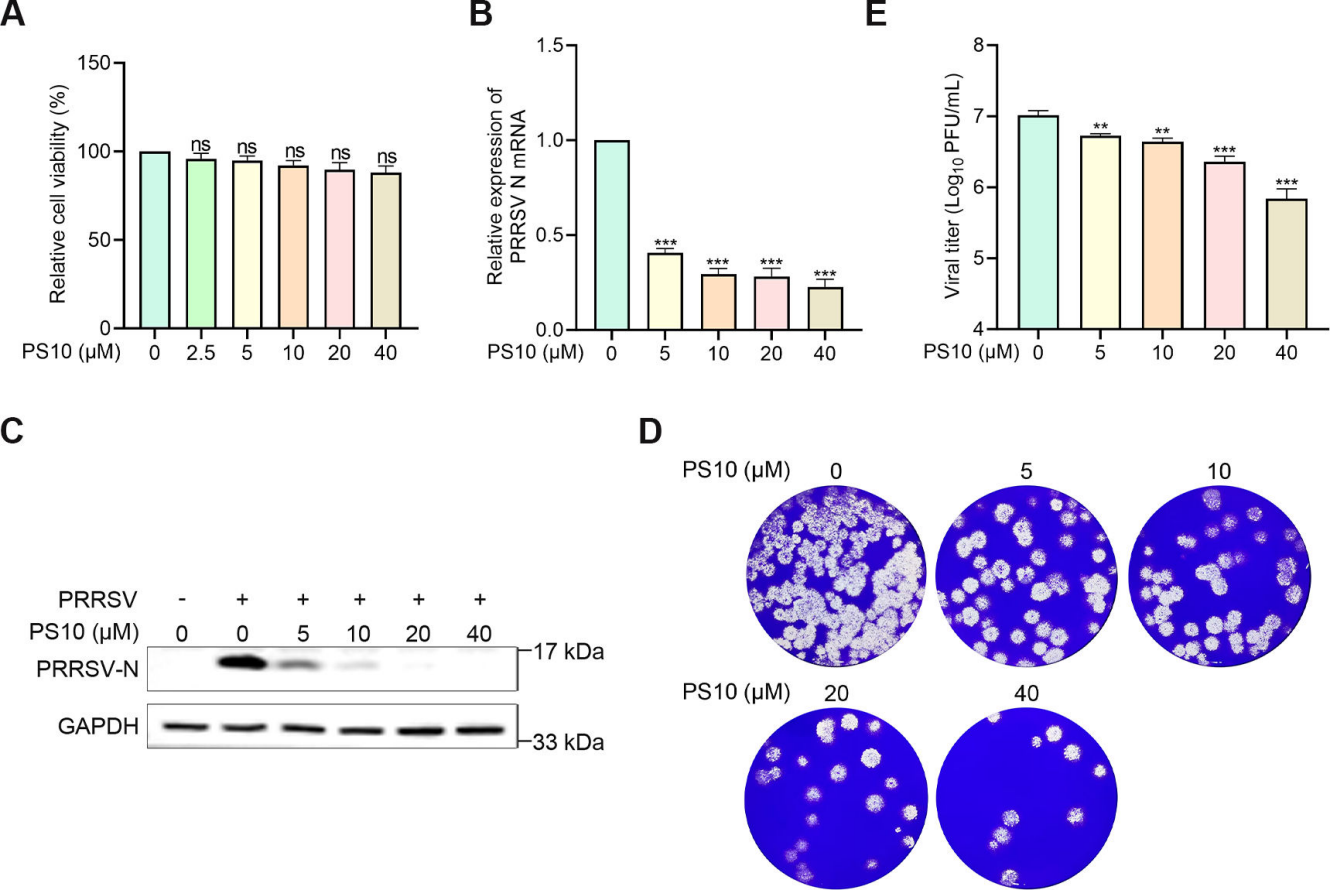
PS10 suppresses PRRSV infection in PAMs. (**A**) The viability of PAMs treated with the indicated concentrations of PS10 for 24 h was assessed using a CCK-8 assay. (**B–E**) PAMs were treated with PS10 for 2 h and then infected with PRRSV JXA1 (MOI = 0.5) for 24 h, after which cells and supernatants were harvested for analysis. Viral N mRNA and protein levels were quantified by RT-qPCR and Western blotting, respectively (**B and C**). Virus titers in the supernatants were determined by plaque assay on Marc-145 cells (**D and E**). ***P < 0.001; **P < 0.01; *P < 0.05; ns: not significant.

Since PAMs are the primary target cells for PRRSV infection, we investigated whether PS10 could also inhibit PRRSV replication in PAMs. Initial cytotoxicity assessment revealed no significant cytotoxic effects of PS10 on PAMs at concentrations ≤ 40 µM (Fig. 3A). Subsequently, we assessed the antiviral effect of PS10 against PRRSV JXA1 infection in PAMs. The results demonstrated that PS10 treatment significantly reduced the viral mRNA levels (Fig. 3B), N protein abundance (Fig. 3C), and progeny virus production (Fig. 3D and E) in a dose-dependent manner, indicating that PS10 can effectively inhibit PRRSV infection in PAMs as well.

### Antiviral efficacy of PS10 against PRRSV infection under different treatment regimens

**Fig 4:**
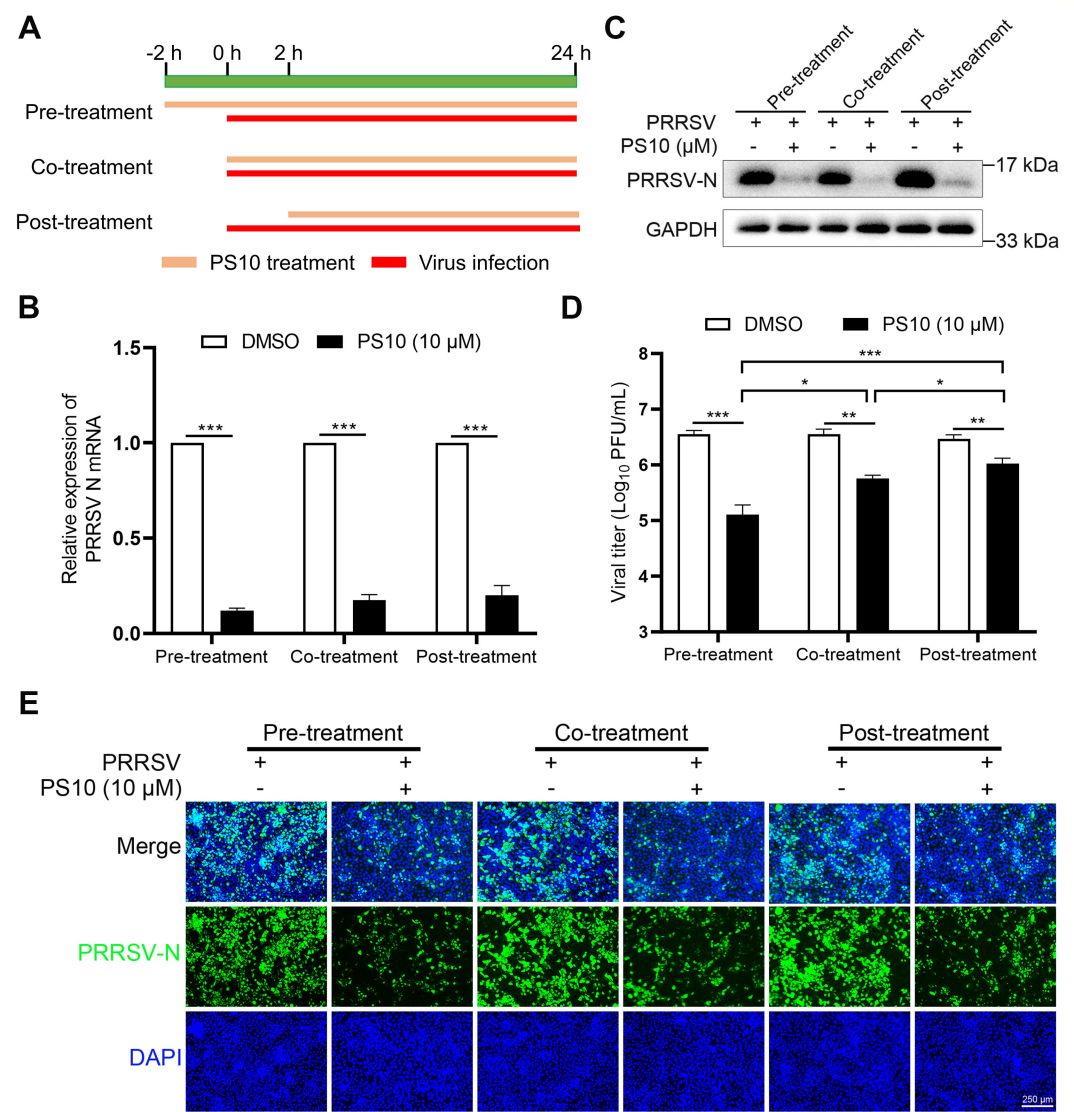
Antiviral efficacy of PS10 against PRRSV infection under different treatment regimens. (**A**) Schematic diagram of the three PS10 treatment protocols. (**B–E**) Marc‐145 cells were treated with PS10 (10 μM) before (pre‐treatment), during (co‐treatment), or after (post‐treatment) infection with PRRSV CH‐1a (MOI = 0.5). Cells and supernatants were harvested 24 hpi for analysis. Viral RNA levels were quantified by RT-qPCR (**B**). Viral N protein expression was assessed by Western blotting (**C**). Virus titers in the supernatants were determined by plaque assay on Marc-145 cells (**D**). Viral N protein fluorescence intensity was analyzed by IFA (**E**). ***P < 0.001; **P < 0.01; *P < 0.05; ns: not significant.

To evaluate the therapeutic potential of PS10 against PRRSV under different treatment modalities, we performed time-of-addition experiments by administering PS10 at three distinct stages: pre-treatment (2 h prior to viral infection), co-treatment (simultaneous with viral inoculation), and post-treatment (2 h after infection) (Fig. 4A). The results demonstrated that all three treatment modalities significantly suppressed both mRNA and protein expression levels of the PRRSV N (Fig. 4B and C), as well as a reduction in viral titers (Fig. 4D) and N protein fluorescence intensity (Fig. 4E). Notably, pre-treatment exhibited the strongest suppression of PRRSV, followed by co-treatment, with post-treatment showing the least effectiveness (Fig. 4B through E). These results demonstrate that PS10 achieves maximal antiviral activity when administered prior to viral infection.

### PS10 does not directly interact with PRRSV virions

**Fig 5:**
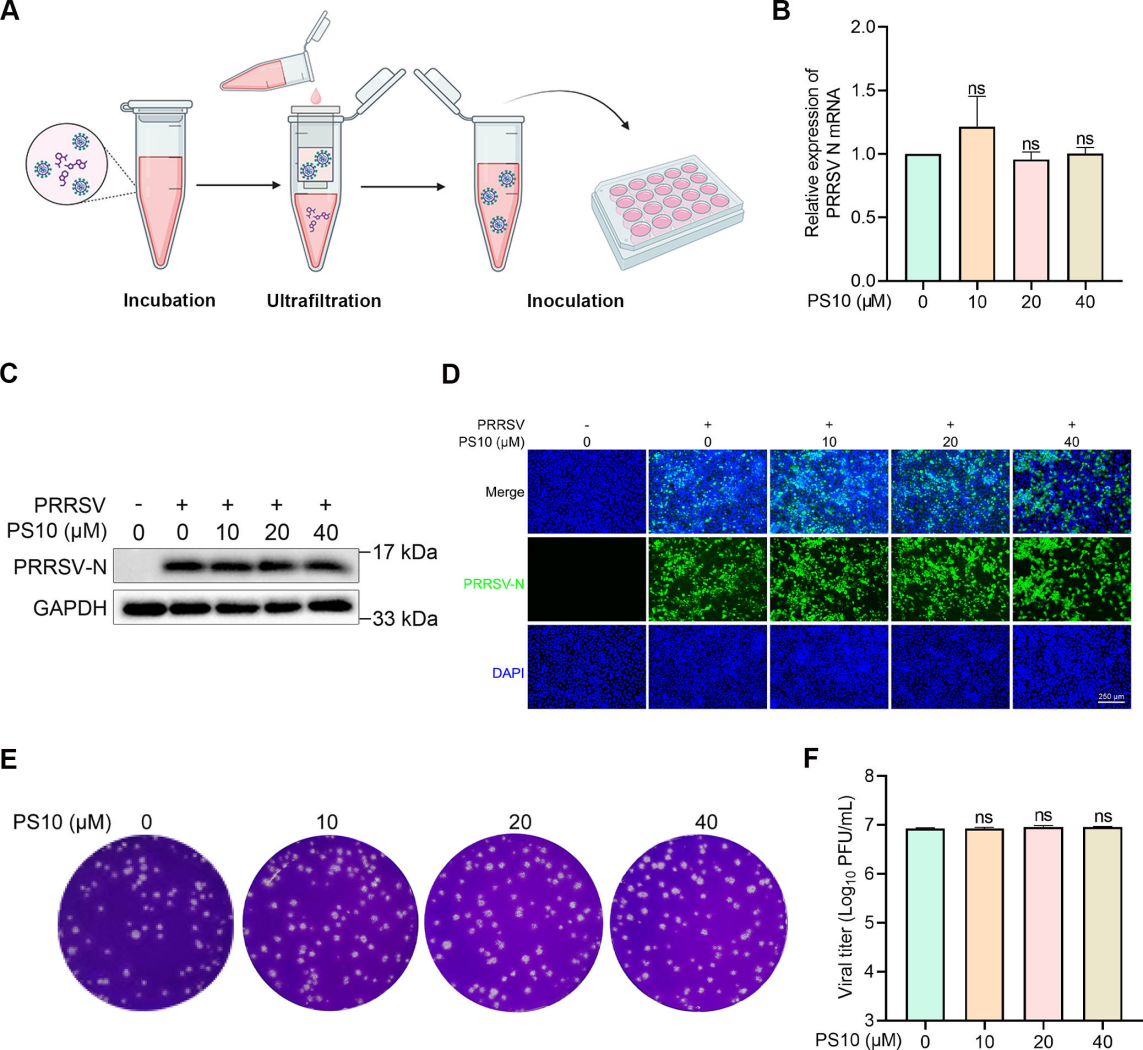
PS10 does not directly interact with PRRSV virions. (**A**) PRRSV CH‐1a virions were incubated with the indicated concentrations of PS10 in basal medium at 37°C for 2 h. The unbound compound was subsequently removed by ultrafiltration. (**B–F**) The recovered virions were resuspended and used to infect Marc‐145 cells. At 24 hpi, cells and supernatants were harvested. Viral N mRNA levels were quantified by RT-qPCR (**B**), and N protein expression was assessed by Western blotting (**C**) and IFA (**D**). Virus titers in the supernatants were determined by plaque assay on Marc-145 cells (**E and F**). ***P < 0.001; **P < 0.01; *P < 0.05; ns: not significant.

Some antiviral drugs inactivate viruses by disrupting virion integrity, thereby reducing the number of infectious particles and impairing viral entry (21). To investigate whether PS10 directly targets PRRSV particles, we incubated the virus with varying concentrations of PS10 in basal medium at 37°C for 2 h. After ultrafiltration to remove unbound PS10, the treated virions were resuspended in fresh medium and used to infect Marc-145 cells (Fig. 5A). Infection efficiency was quantified by measuring viral N protein expression and progeny virus production at 24 hpi. Notably, PRRSV pre-incubated with PS10 showed no significant reduction in infectivity compared to the control group (Fig. 5B through F), indicating that PS10 does not directly interact with or destabilize PRRSV virions. These results suggest that PS10’s antiviral mechanism targets host or viral replication factors rather than structural components of the virus particle.

### PS10 broadly suppresses the replication of diverse PRRSV strains

**Fig 6:**
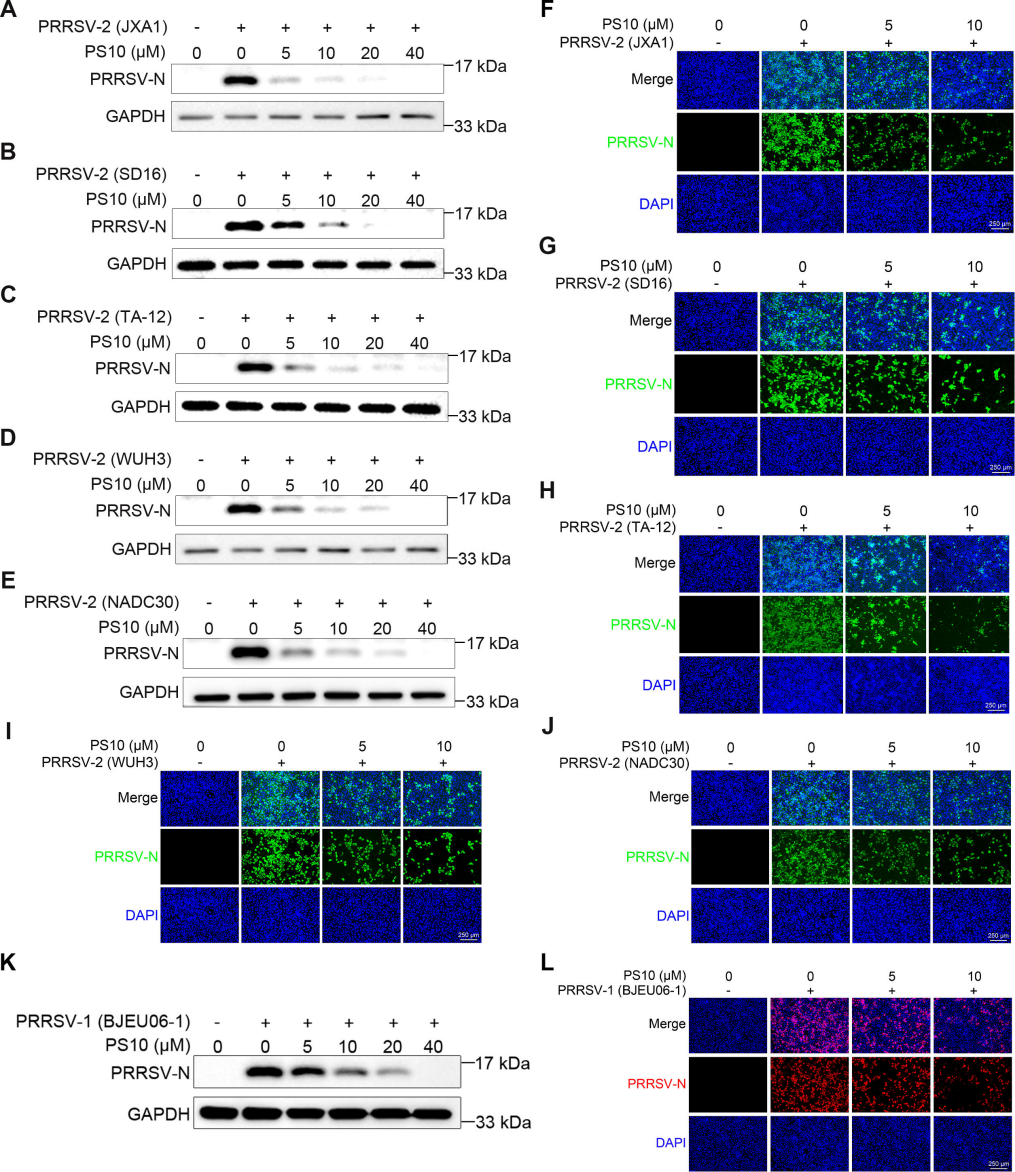
PS10 broadly suppresses the replication of diverse PRRSV strains. (**A–J**) Marc-145 cells were treated with PS10 for 2 h and then infected with PRRSV-2 strains (JXA1, SD16, TA-12, WUH3, and NADC30) at an MOI of 0.5 for 24 h. Samples were subsequently collected and analyzed by Western blotting (**A–E**) and IFA (**F–J**). (**K and L**) Marc-145 cells were treated with PS10 for 2 h and then infected with PRRSV-1 strain BJEU06-1 at an MOI of 0.5. After 24 h, samples were collected and analyzed by Western blotting (**K**) and IFA (**L**).

The substantial genetic diversity among PRRSV strains severely compromises the efficacy of current vaccines and poses significant challenges for disease control. To evaluate whether PS10 broadly inhibits the replication of diverse PRRSV strains, cells were treated with varying doses of PS10 followed by infection with multiple PRRSV-2 isolates (JXA1, SD16, TA-12, WUH3, and NADC30). The results demonstrated that PS10 treatment induced dose-dependent reductions in viral N protein levels across all strains tested (Fig. 6A through E). Consistent with these findings, quantitative fluorescence intensity analysis revealed significantly diminished PRRSV N protein signals (Fig. 6F through J). Furthermore, we evaluated PS10 against the PRRSV-1 strain BJEU06-1. Notably, PS10 also exhibited significant inhibitory activity, markedly reducing the N protein expression of this strain (Fig. 6K and L). Collectively, these results indicate that PS10 exhibits broad-spectrum antiviral activity against diverse PRRSV strains.

### PS10 specifically inhibits the replication stage of the PRRSV life cycle

**Fig 7:**
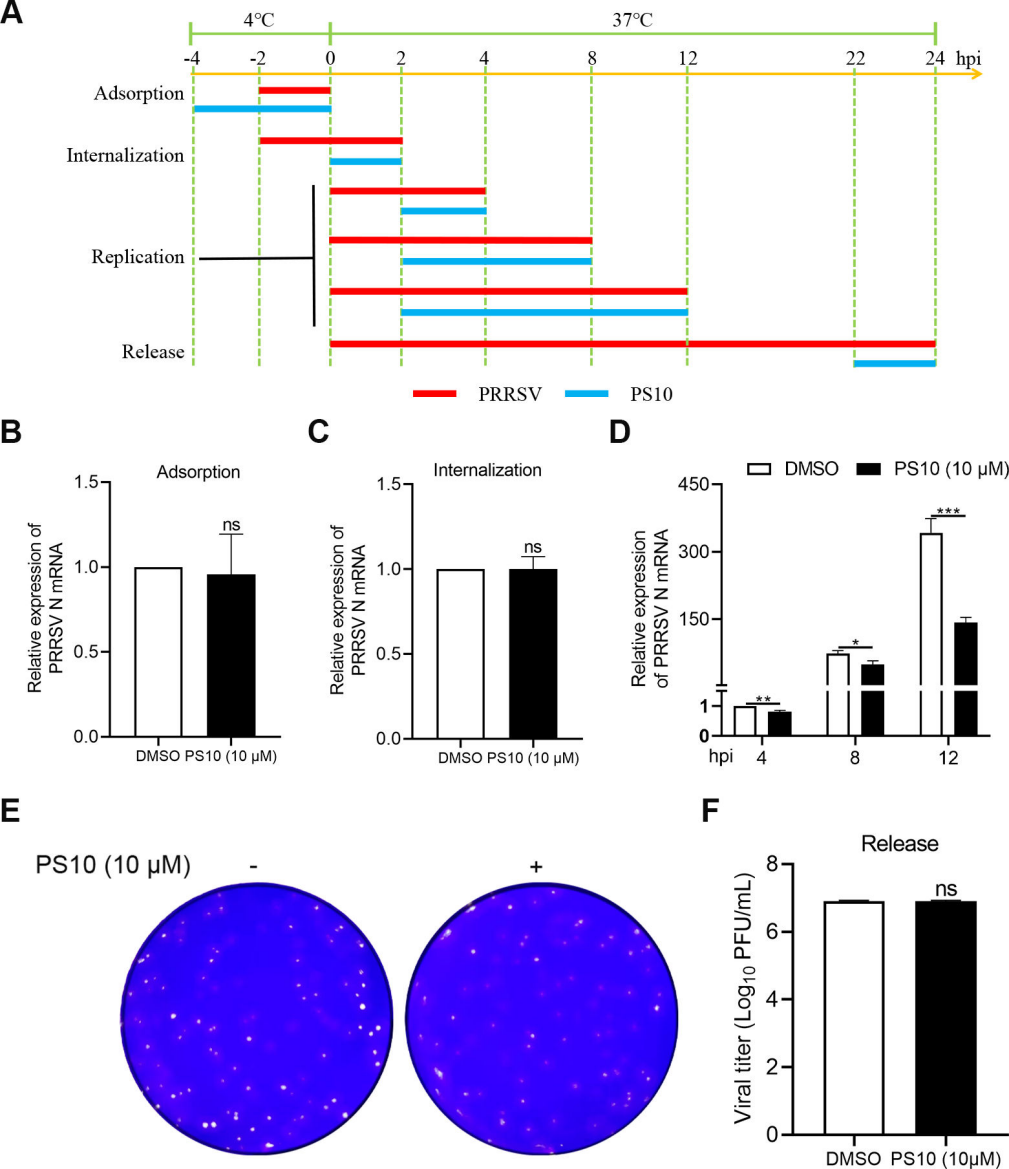
PS10 specifically inhibits the replication stage of the PRRSV life cycle. (**A**) Schematic of the viral binding, entry, replication, and release assays. (**B**) For the binding assay, Marc-145 cells were treated with PS10 (10 μM) at 4°C for 2 h and then infected with the PRRSV CH-1a (MOI = 10) at 4°C for 2 h. Viral N mRNA expression was quantified by RT-qPCR. (**C**) For the internalization assay, Marc-145 cells were infected with the PRRSV CH-1a (MOI = 10) at 4°C for 2 h and then treated with PS10 (10 μM) at 37°C for 2 h. Viral N mRNA expression was quantified by RT-qPCR. (**D**) For the replication assay, Marc-145 cells were infected with the PRRSV CH-1a (MOI = 0.5) at 37°C for 2 h and then treated with PS10 (10 μM) for the indicated durations. Viral N mRNA expression was quantified by RT-qPCR. (**E and F**) For the release assay, Marc-145 cells were infected with the PRRSV CH-1a (MOI = 0.5) at 37°C for 22 h and then treated with PS10 (10 μM) at 37°C for 2 h. Virus titers in the supernatants were determined by plaque assay on Marc-145 cells. ***P < 0.001; **P < 0.01; *P < 0.05; ns: not significant.

The PRRSV life cycle primarily consists of four stages: adsorption, internalization, replication, and release. To investigate the mechanism of PS10 against PRRSV, we systematically examined its effects on each phase of the viral life cycle (Fig. 7A). Quantification of PRRSV N mRNA indicated that PS10 affected neither viral adsorption nor internalization (Fig. 7B and C). Plaque assays further confirmed that PS10 had no impact on the viral release process (Fig. 7E and F). In contrast, viral RNA quantification revealed that PS10 significantly inhibited the replication stage of PRRSV (Fig. 7D). These findings demonstrate that PS10 specifically inhibits the replication stage of PRRSV without affecting viral adsorption, internalization, or release.

### PS10 suppresses PRRSV-induced HSP90 expression

**Fig 8:**
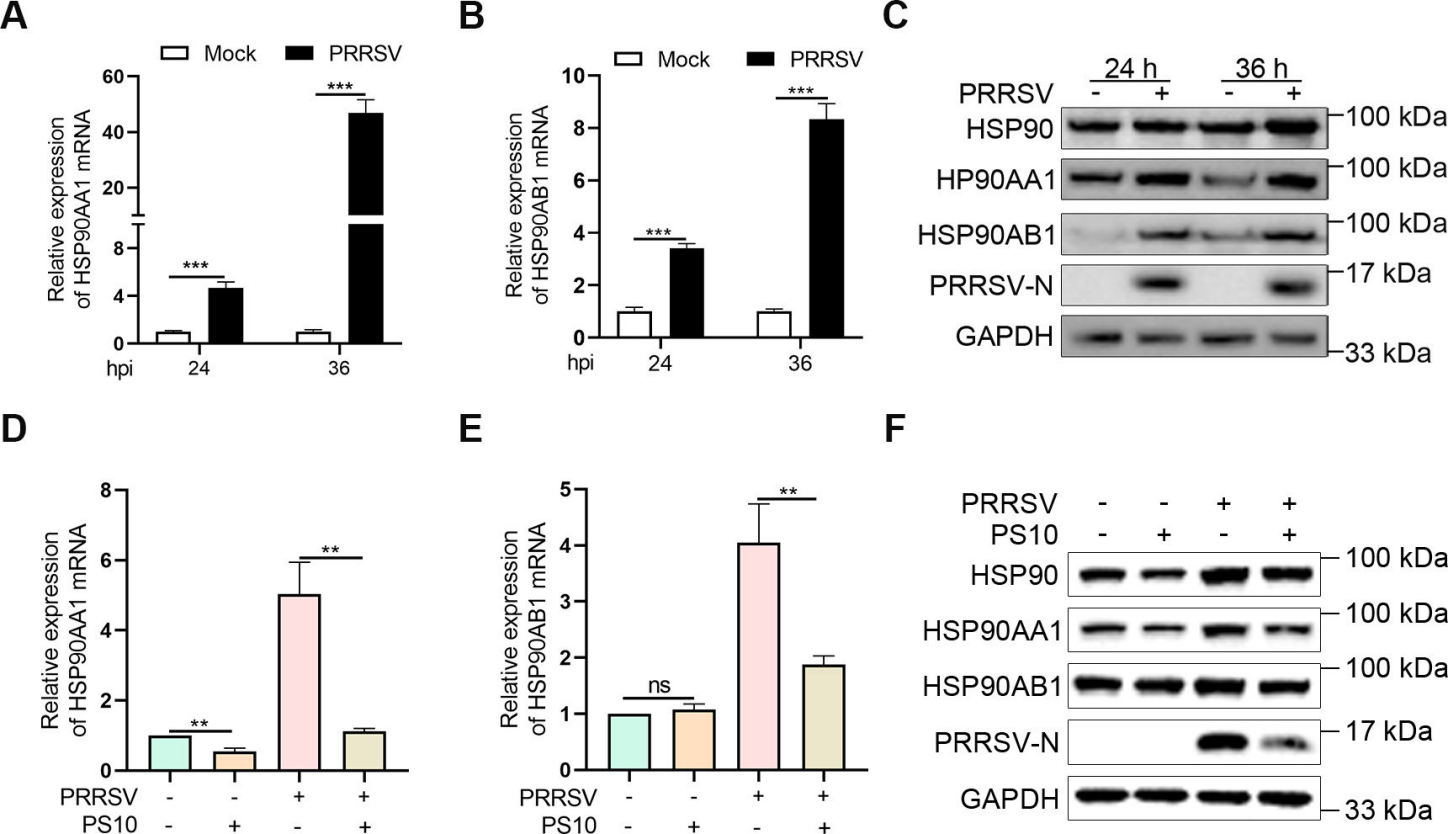
PS10 suppresses PRRSV-induced HSP90 expression. (**A–C**) Marc-145 cells were infected with PRRSV CH-1a (MOI = 0.5) for 24 h and 36 h. HSP90AA1 and HSP90AB1 mRNA levels were quantified by RT-qPCR (**A and B**), and HSP90, HSP90AA1, and HSP90AB1 protein levels were analyzed by Western blotting (**C**). (**D–F**) Marc-145 cells were pretreated with or without PS10 (10 μM) for 2 h and then infected with or without the PRRSV CH-1a (MOI = 0.5) for 24 h. HSP90AA1 and HSP90AB1 mRNA levels were measured by RT-qPCR (**D and E**), and HSP90, HSP90AA1, and HSP90AB1 protein levels were assessed by Western blotting (**F**). ***P < 0.001; **P < 0.01; *P < 0.05; ns: not significant.

Small-molecule compounds can modulate target protein expression through direct binding (17). Previous studies have indicated that PS10 binds to HSP90 (20). Given this binding relationship, it is crucial to explore whether there are other connections between PS10 and HSP90, as these potential connections might have implications for the biological processes involved. Additionally, previous studies have reported that HSP90 is essential for PRRSV replication (22). In light of this, we first examined the effect of PRRSV infection on HSP90 expression. The results demonstrated that PRRSV infection significantly enhanced the transcription of both HSP90AA1 and HSP90AB1 (Fig. 8A and B). Consistent with this, PRRSV infection also markedly upregulated the protein expression levels of total HSP90, HSP90AA1, and HSP90AB1 (Fig. 8C). We further assessed whether PS10 influences PRRSV-induced HSP90 expression. The results indicated that PS10 significantly suppressed the mRNA levels of HSP90AA1 and HSP90AB1 induced by PRRSV infection (Fig. 8D and E). Correspondingly, PS10 also markedly inhibited the PRRSV-induced protein expression of total HSP90, HSP90AA1, and HSP90AB1 (Fig. 8F). These results indicate that PS10 inhibits the up-regulation of HSP90 expression caused by PRRSV.

### PS10 significantly restricts PRRSV proliferation by targeting and suppressing HSP90 function

**Fig 9:**
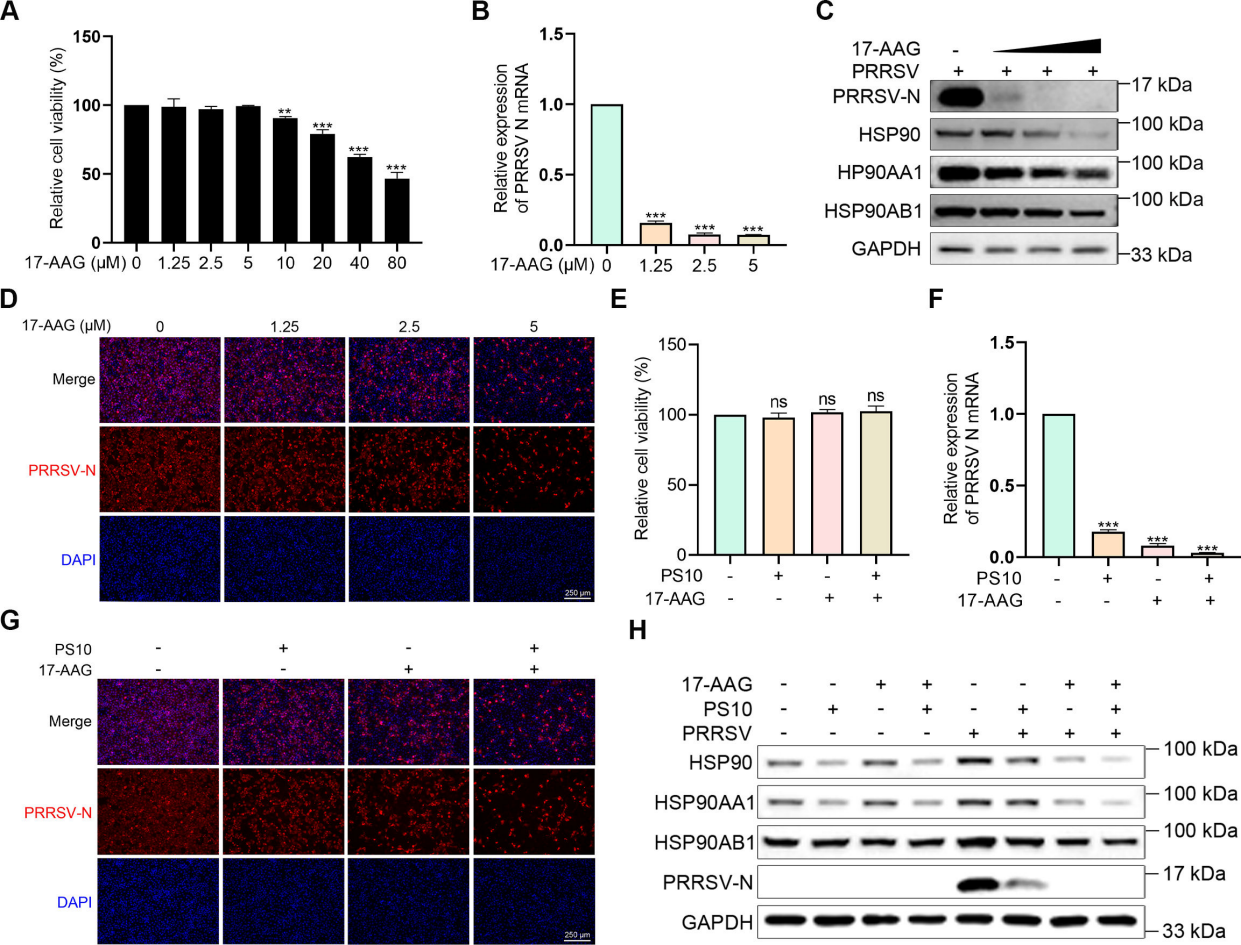
PS10 significantly restricts PRRSV proliferation by targeting and suppressing HSP90 function. (**A**) Viability of Marc-145 cells treated with the indicated concentrations of 17-AAG for 24 h was assessed using a CCK-8 assay. (**B–D**) Marc-145 cells were treated with 17-AAG for 2 h and then infected with PRRSV CH-1a (MOI = 0.5) for 24 h. Subsequently, samples were collected and analyzed by RT-qPCR (**B**), Western blotting (**C**), and IFA (**D**). (**E**) Viability of Marc-145 cells treated with PS10 (5 μM), 17-AAG (2.5 μM), or their combination for 24 h was assessed by CCK-8 assay. (**F–H**) Marc-145 cells were treated with PS10 (5 μM), 17-AAG (2.5 μM), or their combination for 2 h, and then infected with PRRSV CH-1a (MOI = 0.5) for 24 h. Mock-infected cells served as a control. Samples were collected and analyzed by RT-qPCR (**F**), IFA (**G**), and Western blotting (**H**). ***P < 0.001; **P < 0.01; *P < 0.05; ns: not significant.

Given the critical role of HSP90 in the life cycle of various viruses, we investigated whether PS10 affects viral replication by modulating HSP90 expression. We first assessed the impact of 17-AAG, a known HSP90 inhibitor, on the viability of Marc-145 cells. The results showed that 17-AAG concentrations below 10 μM had no significant effect on cell viability (Fig. 9A). We further evaluated the effect of 17-AAG on PRRSV replication. As the concentration of 17-AAG increased, both the PRRSV genomic copy number and the level of the viral N protein decreased in a dose-dependent manner (Fig. 9B through D). Concurrently, the protein levels of total HSP90, HSP90AA1, and HSP90AB1 were also reduced dose-dependently (Fig. 9C). To determine whether PS10 acts similarly, we compared its effects with 17-AAG, alone and in combination. Co-treatment did not affect cell viability (Fig. 9E). Notably, combined treatment with 17-AAG and PS10 led to the most substantial suppression of HSP90, HSP90AA1, and HSP90AB1 protein expression, and accordingly, the strongest inhibition of PRRSV replication (Fig. 9F through H). These results demonstrate that PS10 inhibits PRRSV replication by downregulating HSP90 expression.

### PS10 suppresses the production of pro-inflammatory cytokines by downregulating HSP90 expression

**Fig 10:**
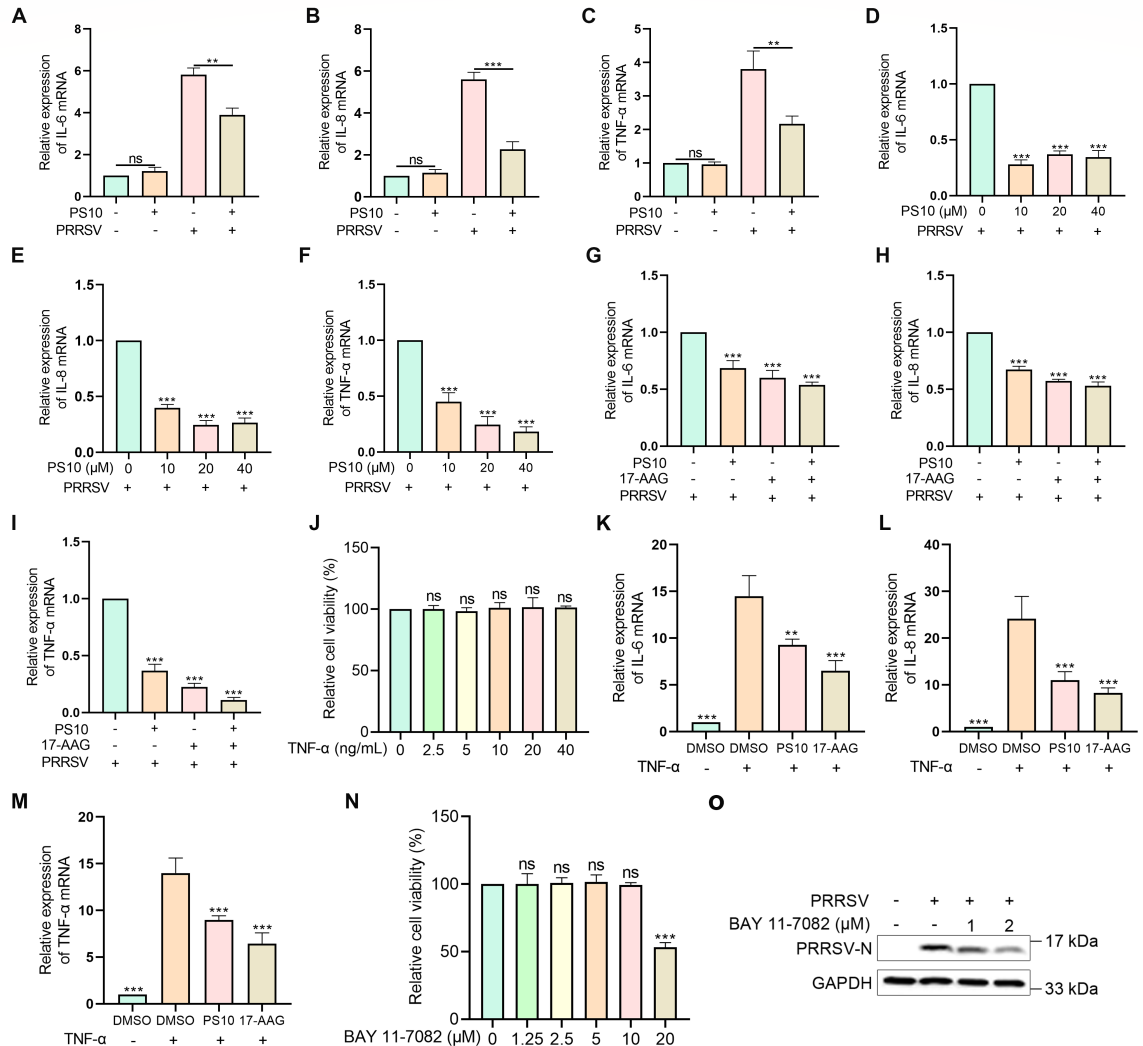
PS10 suppresses the production of pro-inflammatory cytokines by downregulating HSP90 expression. (**A–C**) Marc-145 cells were pretreated with or without PS10 (10 μM) for 2 h and then infected with or without the PRRSV CH-1a (MOI = 0.5) for 24 h. IL-6, IL-8, and TNF-α mRNA levels were measured by RT-qPCR. (**D–F**) Marc-145 cells were treated with different concentrations of PS10 for 2 h and then infected with the PRRSV CH-1a (MOI = 0.5) for 24 h. IL-6, IL-8, and TNF-α mRNA levels were measured by RT-qPCR. (**G–I**) Marc-145 cells were treated with PS10 (5 μM), 17-AAG (2.5 μM), or their combination for 2 h and then infected with PRRSV CH-1a (MOI = 0.5) for 24 h. IL-6, IL-8, and TNF-α mRNA levels were measured by RT-qPCR. (**J**) Viability of HeLa cells treated with indicated concentrations of TNF-α for 24 h was assessed by CCK-8 assay. (**K–M**) HeLa cells were treated with PS10 (40 μM) or 17-AAG (1 μM) for 12 h, followed by stimulation with TNF-α (10 ng/mL) for an additional 12 h. mRNA levels of IL-6, IL-8, and TNF-α were quantified by RT-qPCR. (**N**) Viability of Marc-145 cells treated with indicated concentrations of BAY 11-7082 for 24 h was assessed by CCK-8 assay. (**O**) Marc-145 cells were treated with different concentrations of BAY 11-7082 for 2 h and then infected with the PRRSV CH-1a (MOI = 0.5) for 24 h. N protein expression was analyzed by Western blotting. ***P < 0.001; **P < 0.01; *P < 0.05; ns: not significant.

HSP90 modulates inflammatory responses through multiple mechanisms (23). Given that PRRSV infection induces a storm of inflammatory cytokines in the lungs, contributing to respiratory symptoms, we investigated whether PS10 affects the production of inflammatory cytokines by regulating HSP90 expression. We first examined the effect of PS10 on PRRSV-induced expression of inflammatory factors. The results showed that PS10 significantly suppressed the mRNA expression of IL-6, IL-8, and TNF-α (Fig. 10A through C). Moreover, with increasing doses of PS10, the expression of IL-6, IL-8, and TNF-α mRNA also significantly decreased (Fig. 10D through F). Notably, the combined use of 17-AAG and PS10 inhibited the mRNA levels of IL-6, IL-8, and TNF-α more effectively than either compound alone (Fig. 10G through I). To rule out the possibility that the reduction in pro-inflammatory cytokines was secondary to decreased PRRSV infection, we tested PS10 and 17-AAG in uninfected HeLa cells stimulated with TNF-α. At a non-cytotoxic concentration, TNF-α induced cytokine production, which was significantly suppressed by both compounds (Fig. 10J through M). These results indicate that PS10 can inhibit inflammatory factors by suppressing HSP90 expression, rather than solely as a consequence of reduced PRRSV infection. We then asked whether suppressing inflammation itself affects PRRSV replication. Using the NF-κB inhibitor BAY 11-7082, we observed a dose-dependent reduction in PRRSV N-protein expression (Fig. 10N and O). Together, these findings demonstrate that PS10 inhibits the production of pro-inflammatory cytokines by downregulating HSP90 expression. This immunomodulatory action cooperates with direct antiviral mechanisms to suppress PRRSV infection.

### PS10 specifically decreases the abundance of PRRSV nsp2, nsp3, nsp10, and nsp11

**Fig 11:**
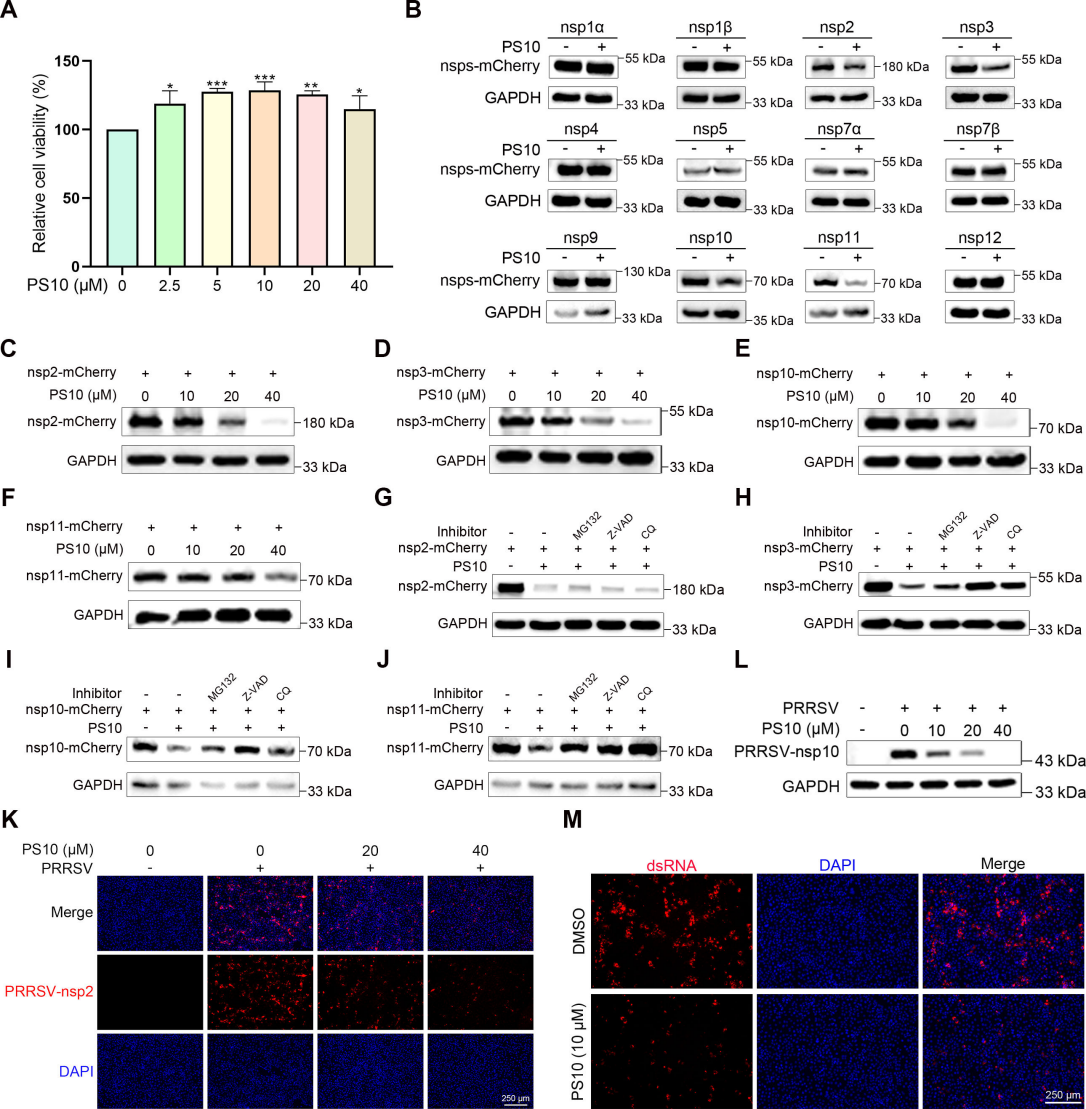
PS10 specifically reduces the abundance of PRRSV nsp2, nsp3, nsp10, and nsp11. (**A**) Viability of HEK-293T cells treated with the indicated concentrations of PS10 for 24 h was assessed using a CCK-8 assay. (**B**) HEK293T cells were transfected with plasmids encoding PRRSV nsps-mCherry. At 6 h post-transfection, cells were treated with PS10 (40 μM) or DMSO and harvested at 24 h for Western blot analysis of nsp protein levels. (**C–F**) HEK293T cells transfected with plasmids encoding individual nsp2, nsp3, nsp10, or nsp11 were treated with the indicated concentrations of PS10 at 6 h post-transfection. Cells were harvested at 24 h for Western blot analysis. (**G–J**) HEK293T cells were transfected with plasmids encoding individual nsp2, nsp3, nsp10, or nsp11. Cells were treated with PS10 (40 μM) or DMSO at 6 h and with corresponding inhibitors at 12 h post-transfection. All cells were harvested at 24 h for Western blot analysis. (**K and L**) Marc-145 cells were treated with PS10 for 2 h and then infected with PRRSV CH-1a (MOI = 0.5). After 24 h, samples were collected and analyzed by Western blotting (**K**) and IFA (**L**). (**M**) Marc-145 cells were pretreated with PS10 (10 μM) for 2 h and then infected with the PRRSV CH-1a (MOI = 0.5) for 12 h. Viral dsRNA was detected by IFA. ***P < 0.001; **P < 0.01; *P < 0.05; ns: not significant.

Our study demonstrates that PS10 inhibits the replication stage of the PRRSV life cycle. Given the crucial roles played by PRRSV nsps in viral replication, we investigated whether PS10 affects PRRSV replication by influencing the stability of these nsps. To rule out potential cytotoxic effects of PS10 on HEK293T cells, we first assessed its impact on cell viability. The results showed that PS10 at concentrations up to 40 μM did not affect HEK293T cell viability (Fig. 11A). Further investigation revealed that PS10 treatment specifically and significantly reduced the abundance of nsp2, nsp3, nsp10, and nsp11 proteins without affecting the expression of other nsps (Fig. 11B). Moreover, the inhibitory effect of PS10 on these proteins exhibited a clear dose-dependent manner, with higher concentrations leading to more pronounced suppression (Fig. 11C through F). In eukaryotic cells, protein degradation occurs primarily through three major pathways: the proteasome, autophagy, and apoptosis. To examine whether PS10-mediated reduction of nsps involves these mechanisms, we treated cells with specific inhibitors targeting the macroautophagy-lysosomal pathway (CQ), the ubiquitin-proteasome system (MG132), or caspase-mediated apoptotic (Z-VAD). We found that none of the inhibitors prevented PS10-induced reduction of nsp2 (Fig. 11G). In contrast, the reduced abundance of nsp3, nsp10, and nsp11 was partially rescued by these inhibitors (Fig. 11H through J). PS10 also reduced nsp2 and nsp10 levels during PRRSV infection (Fig. 11K and L). Given that PS10 compromises the stability of key replicase subunits, including the essential replication-transcription complex (RTC) components nsp2 and nsp3, we hypothesized that it would interfere with the replication phase of the PRRSV life cycle. As double-stranded RNA (dsRNA) serves as a replication intermediate and is indicative of active viral replication, we investigated the impact of PS10 on dsRNA synthesis. Our results showed that treatment with PS10 significantly suppressed the formation of PRRSV dsRNA (Fig. 11M). Taken together, these results indicate that PS10 inhibits PRRSV replication by reducing the abundance of key nsps (nsp2, nsp3, nsp10, and nsp11).

### PS10 binds to PRRSV nsp2, nsp3, nsp10, and nsp11

**Fig 12:**
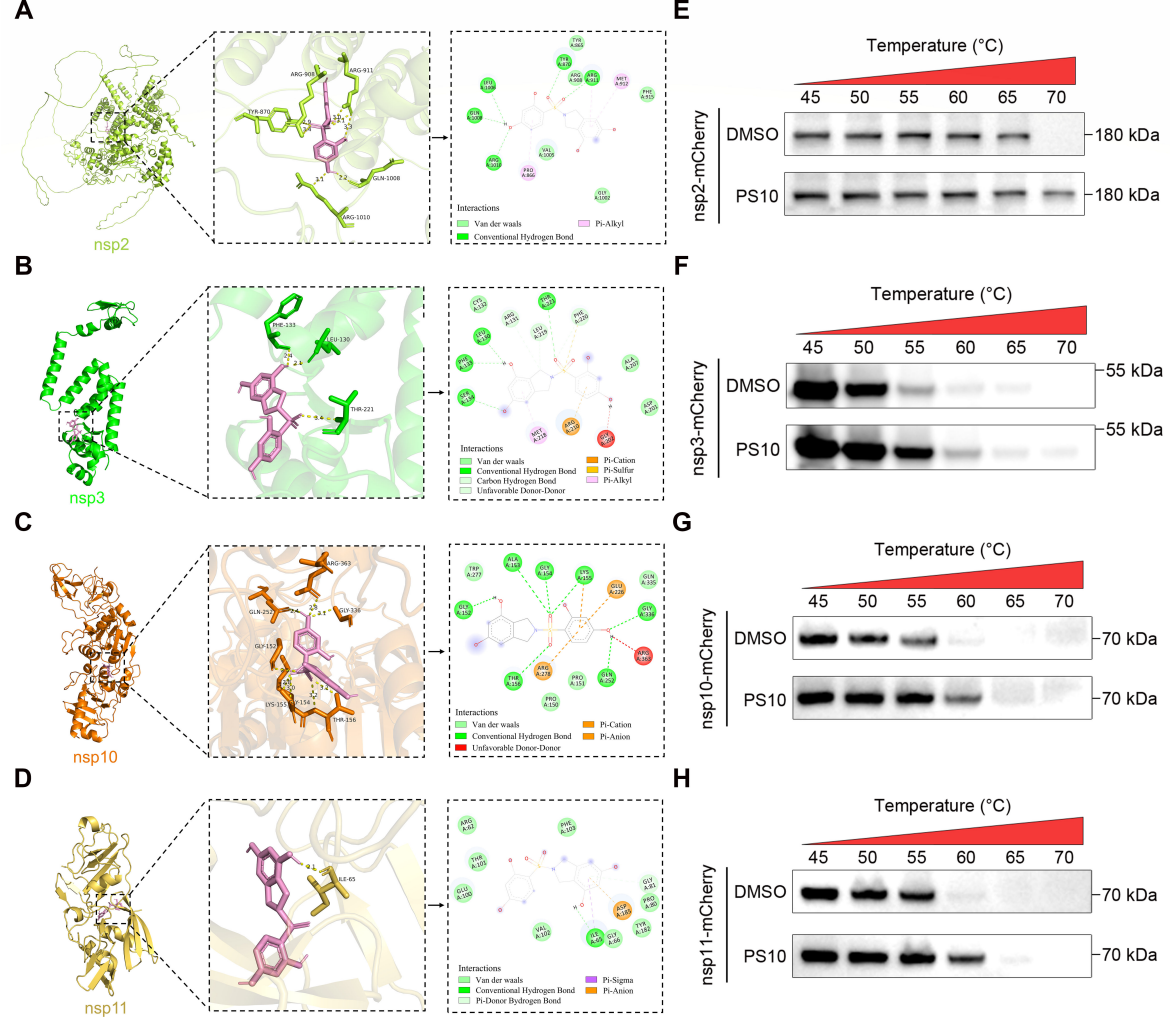
PS10 binds to PRRSV nsp2, nsp3, nsp10, and nsp11. (**A–D**) Ligand structures and protein structures were obtained from PubChem and AlphaFold3, respectively. Molecular docking was performed using CB-Dock2, and the results were visualized with Discovery Studio and PyMOL. (**E–H**) The cells were transfected with plasmids encoding viral proteins for 24 h, followed by lysis. Subsequently, the lysate was incubated with PS10 (40 μM) at 37°C for 30 min. The mixture was then transferred to a PCR machine and heated at different temperature gradients for 3 min, followed by three cycles of freeze-thawing in liquid nitrogen. After centrifugation, the supernatant was collected for SDS-PAGE analysis.

Given that PS10 affects the stability of nsp2, nsp3, nsp10, and nsp11, we hypothesized that PS10 might interact with these nsps. To evaluate this possibility, we first performed computational molecular docking to analyze potential interactions between PS10 and each target protein. The docking results indicated favorable binding interactions between PS10 and nsp2, nsp3, nsp10, and nsp11 (Fig. 12A through D). We further validated these interactions using cellular thermal shift assay (CETSA). Protein extracts from HEK293T cells expressing individual nsps were treated with PS10 (40 μM) or DMSO and subjected to a heat pulse. The results demonstrated that PS10 significantly enhanced the thermal stability of exogenously expressed nsp2, nsp3, nsp10, and nsp11 and delayed their heat-induced degradation (Fig. 12E through H). Collectively, these findings provide direct evidence that PS10 can physically bind to nsp2, nsp3, nsp10, and nsp11.

## DISCUSSION

The increasing threat of emerging and re-emerging animal infectious diseases jeopardizes global livestock production, food security, and the sustainability of animal-sourced food systems (24). In China, pork plays a vital role in national food security (25). Porcine viral diseases persistently challenge swine industry development, and among these, PRRSV has become particularly detrimental due to its high genetic diversity, frequent mutations, and recombination events, which undermine current vaccine efficacy (26). Thus, effective therapeutic interventions against PRRSV infection are urgently needed. While vaccination remains a primary strategy, its limitations highlight the critical need for drug therapy (27, 28). Previous studies have identified several compounds with anti-PRRSV activity *in vitro* and *in vivo* (29–31). To expand the antiviral candidate pool, we performed systematic screening and identified PS10 as a novel inhibitor. PS10 showed low cytotoxicity in Marc-145 cells and PAMs and displayed potent anti-PRRSV activity *in vitro*. Given its low-cost profile and potent efficacy at low doses, PS10 is a promising candidate for targeted anti-PRRSV therapy. We observed that its inhibitory efficacy was somewhat lower in PAMs than in Marc-145 cells, likely due to inherent biological differences between cell types and the use of distinct PRRSV strains. Following PS10 treatment, we noted heterogeneous plaque sizes. High-throughput sequencing revealed a PS10-induced mutation in the PRRSV genome, which may attenuate viral replication or represent an adaptive, resistance-conferring change (data not shown). Certain antiviral agents can suppress replication by increasing viral mutagenesis (32).

Treatment regimen studies indicated that pre-treatment with PS10 had the strongest antiviral effect, while post-treatment was least effective. Time-course experiments further showed that PS10’s inhibitory capacity diminished during later infection stages, suggesting prophylactic administration may be the optimal strategy for PRRSV control. Although some antiviral compounds act by directly inactivating viral particles (33), we found that PS10 does not directly interact with the virion. Given PRRSV’s genetic diversity, we evaluated PS10 against multiple strains and observed broad-spectrum inhibitory activity. As an enveloped virus, PRRSV progresses through attachment, internalization, replication, and release (34, 35). Our mechanistic studies revealed that PS10 specifically disrupts the replication stage, likely by modulating host factors required for viral replication. Previous studies indicate HSP90 is a binding target of PS10 (20). HSP90 is a key molecular chaperone that supports the folding, stabilization, and nuclear transport of multiple viral proteins (36). It has been demonstrated that Carrimycin interacts with transmembrane protein B (TMEM41B) and induces its degradation via the proteasomal pathway (17). Based on this precedent, we investigated whether PS10 similarly modulates HSP90 expression. Our results showed that PS10 suppressed the PRRSV-induced expression of HSP90 and thereby inhibited viral replication. This aligns with the established role of HSP90 in the replication of diverse viruses, including PRRSV (22), African swine fever virus (ASFV) (37), herpes simplex virus type I (HSV-1) (38), and bluetongue virus (BTV) (39).

During PRRSV infection, the virus activates NF-κB to upregulate pro-inflammatory cytokines, contributing to the systemic hyperinflammatory response characteristic of PRRSV pathogenesis (40). Notably, HSP90 stabilizes IKK-β to sustain NF-κB signaling (41) and facilitates NLRP3 inflammasome activation (42). Consistent with this, the HSP90 inhibitor 17-AAG suppresses inflammatory cytokine production (43). We therefore hypothesized that PS10 might attenuate PRRSV-induced inflammation by suppressing HSP90 expression. Indeed, PS10 significantly inhibits pro-inflammatory cytokine production during PRRSV infection. In uninfected cells stimulated with TNF-α, both PS10 and 17-AAG reduced cytokine expression, confirming that the anti-inflammatory effect of PS10 is mediated, at least in part, through HSP90 downregulation and is not merely due to reduced viral infection. These results suggest that PS10 may also exert antiviral activity against other viruses by downregulating HSP90 expression.

Previous studies have established that multiple antiviral compounds inhibit viral replication by directly targeting viral proteins (44–46). In PRRSV, nsps play critical roles in viral replication and pathogenesis (47, 48). Since PS10 was found to specifically block the replication stage of PRRSV, we investigated its effects on nsps expression. PS10 significantly reduced levels of nsp2, nsp3, nsp10, and nsp11. Notably, the decrease in nsp3, nsp10, and nsp11 could be rescued by CQ, MG132, or Z-VAD, whereas nsp2 degradation was not reversed by any of these treatments, suggesting a distinct degradation mechanism. PS10 also suppressed nsp2 and nsp10 protein expression during infection. These nsps perform key functions: nsp2 possesses PLP2 protease activity (49), nsp3 is a transmembrane protein essential for the replication-transcription complex (50), nsp10 functions as a helicase (11), and nsp11 exhibits endoribonuclease activity (14). Furthermore, CETSA confirmed interactions between PS10 and these nsps, a finding that was corroborated by molecular docking analysis. This binding may explain PS10’s ability to suppress the expression of these proteins. These findings align with prior reports demonstrating that small molecules can exert antiviral effects through interactions with viral proteins (51–53). Interestingly, we also observed that PS10 treatment enhanced the viability of HEK-293T cells without affecting Marc-145 cells or PAMs. This cell type-specific pro-survival effect may be related to differences in basal metabolic or stress states and warrants further investigation.

**Fig 13:**
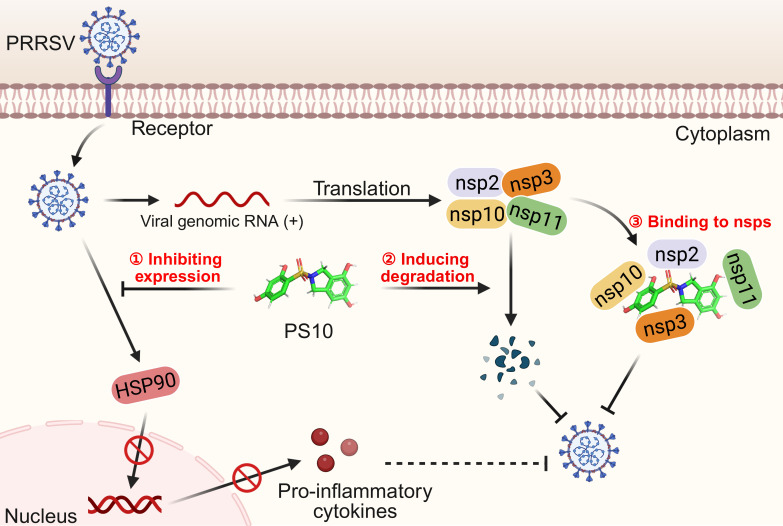
Schematic model illustrating the mechanism of PS10-mediated inhibition of PRRSV replication. Following receptor binding and entry into the host cell, PS10 inhibits the replication phase of the PRRSV life cycle. Specifically, (ⅰ) PS10 significantly suppresses PRRSV infection-induced expression of HSP90, thereby impairing viral propagation and attenuating pro-inflammatory cytokine production. (ⅱ) PS10 can also selectively target and degrade multiple viral nsps, including nsp2, nsp3, nsp10, and nsp11. This targeted degradation constitutes the key mechanism underlying its antiviral activity. (ⅲ) Furthermore, PS10 binds to these viral proteins, which may contribute to its inhibition of PRRSV replication.

In conclusion, our study demonstrates for the first time that PS10 functions as a critical antiviral agent, specifically against PRRSV. Its mechanism involves suppressing viral proliferation and mitigating inflammatory responses by downregulating HSP90 expression. Furthermore, PS10 interacts with PRRSV nsp2, nsp3, nsp10, and nsp11, which may contribute to its specific inhibition of these proteins (Fig. 13). These findings highlight PS10 as a promising lead compound for anti-PRRSV therapy. Moreover, its dual targeting of host and viral factors suggests potential broad-spectrum antiviral applications.

## MATERIALS AND METHODS

### Cells and viruses

HEK293T cells, Marc-145 cells, and HeLa cells were maintained in Dulbecco’s modified Eagle’s medium (DMEM; Bio-Channel, BC20250708) supplemented with 10% fetal bovine serum (FBS; ExCell Bio, FSP500). Primary porcine alveolar macrophages, the natural target cells for PRRSV infection, were isolated from 4-week-old PRRSV-negative piglets using established protocols and cultured in RPMI 1640 medium (Gibco) containing 10% FBS. The PRRSV-2 strains JXA1 (FJ548855.1), SD16 (JX087437.1), TA-12 (HQ416720.1), WUH3 (HM853673.2), NADC30 (MH500776), and CH-1a (AY032626.1), and the PRRSV-1 strain BJEU06-1 (GU047344.1) were used in this study. All viral stocks were propagated and titrated in Marc-145 cells.

### Reagents and antibodies

The small-molecule PS10 (HY-121744), MG132 (HY-13259), chloroquine (CQ; HY-17589A), Z-VAD (HY-164388), and the NF-κB inhibitor BAY 11-7082 (HY-13453) were purchased from MedChemExpress. Tanespimycin (17-AAG; A220462) was obtained from AmBeed. Recombinant human TNF-α (300-01A) was purchased from Thermo Fisher Scientific. 4ʹ,6-diamidino-2-phenylindole, dihydrochloride (DAPI; C0065, 1:1,000) was acquired from Solarbio, and polyethylenimine (PEI; G1802) was purchased from Servicebio. Anti-PRRSV N monoclonal antibody (JN0401, 1:3,000) was sourced from MEDIAN Diagnostics (Korea). Anti-GAPDH (60004-1-Ig, 1:5,000), Anti-mCherry-tag (26765-1-AP, 1:2,000), HRP-conjugated anti-mouse (SA00001-1, 1:10,000), and anti-rabbit (SA00001-2, 1:10,000) antibodies were acquired from Proteintech Group. Alexa Fluor 488-conjugated goat anti-mouse (4408, 1:1,000) antibodies, as well as Alexa Fluor 555-conjugated goat anti-mouse (4409, 1:1,000) antibodies, were purchased from Cell Signaling Technology. Furthermore, anti-HSP90 (F0283, 1:1,000), anti-HSP90AA1 (F0889, 1:1,000), and anti-HSP90AB1 (F1132, 1:1,000) were sourced from Selleck Chemicals.

### Cytotoxicity assay

Cell viability was determined using the CCK-8 assay (GlpBio, GK10001). Marc-145 cells or PAMs were seeded in 96-well plates and treated with specified compounds for 24 h. After treatment, 10 μL of CCK-8 reagent was added per well, followed by 1-h incubation. Absorbance was measured at 450 nm using a microplate reader.

### RNA extraction and quantitative real-time PCR

Total RNA was extracted from Marc-145 cells or PAMs using TRIeasy Reagent (YEASEN, 10,606ES60) and reverse transcribed into cDNA with ABScript Neo RT Master Mix for qPCR with gDNA Remover (ABclonal, RK20433). Quantitative real-time PCR was conducted using BrightCycle Blue Universal SYBR Green qPCR Mix with UDG (ABclonal, RK21220) on the QuantStudio 3 Real-Time PCR system (Thermo Fisher Scientific). Relative gene expression was normalized to GAPDH (for Marc-145 or HeLa cells) or HPRT1 (for PAMs) and calculated using the 2^−ΔΔCT^ method. All primer sequences are listed in Table S1.

### Western blotting

Cells were lysed in RIPA buffer (Beyotime, P0013B) containing 1 mM PMSF (Solarbio, P0100) on ice for 30 min. Lysates were separated by SDS-PAGE and transferred to PVDF membranes (Merck Millipore, ISEQ00010). After blocking with 5% skim milk (Beyotime, P0216) for 2 h at room temperature, membranes were incubated with primary antibodies overnight at 4°C. Following three washes, membranes were probed with HRP-conjugated secondary antibodies for 1 h at room temperature. Protein signals were detected using an ECL kit (MeilunBio, MA0186-3).

### Indirect IFA

Cells were fixed with 4% paraformaldehyde (Beyotime, P0099) for 15 min at room temperature, followed by permeabilization with 0.5% Triton X-100 (Beyotime, P0096) for 5 min. After three PBS washes, non-specific binding was blocked with 1% BSA (Beyotime, ST2249) for 1 h at room temperature. Primary antibody incubation was performed overnight at 4°C, followed by three PBS washes and 1-h incubation with fluorescent secondary antibodies at room temperature. Nuclei were counterstained with DAPI for 5 min before imaging with an inverted fluorescence microscope (Nikon ECLIPSE Ti2).

### Viral titration by plaque assay

Marc-145 cells in six-well plates were infected with 10-fold serially diluted PRRSV for 1 h (with gentle agitation every 15 min). After removing the inoculum and washing with PBS, cells were overlaid with DMEM containing 1% low-melting agarose and 2% FBS. Following solidification, plates were incubated at 37°C with 5% CO₂ for 72 h. Plaques were visualized by crystal violet staining after agarose removal.

### Direct PRRSV-PS10 interaction

PRRSV was incubated with increasing concentrations of PS10 for 2 h at 37°C to evaluate direct virion interaction. Virus-compound complexes were isolated by centrifugal filter devices (Pall, OD100C33). After three washes with basal medium to remove unbound PS10, purified virions were resuspended and inoculated onto Marc-145 cells for 24-h infectivity analysis.

### Plasmid transfection and treatment

The plasmids expressing the nsps of PRRSV were stored in our laboratory. HEK-293T cells were seeded into 12-well plates. When the cell density reached approximately 80%, these plasmids were transfected into the cells using PEI. Six hours post-transfection, the cells were treated with or without PS10. Samples were collected at 24 h post-transfection for western blotting analysis.

### Cellular thermal shift assay

HEK293 cells transfected with the corresponding plasmids for 24 h were lysed in NP-40 Lysis Buffer (Beyotime, P0013F). Aliquots (50 μL) of lysate were incubated with or without PS10 (40 μM) at 37°C for 30 min. Samples were then divided and heated at 45°C, 50°C, 55°C, 60°C, 65°C, and 70°C for 3 min, followed by three cycles of freezing in liquid nitrogen and thawing at 25°C. After being centrifuged at 12,000 × *g* for 20 min at 4°C, supernatants were carefully transferred to new tubes, mixed with loading buffer, boiled at 100°C for 10 min, and finally analyzed by western blotting.

### Molecular docking

To investigate protein-small molecule interactions, molecular docking was performed. Ligand structures were acquired from PubChem (https://pubchem.ncbi.nlm.nih.gov), and protein structures were predicted using AlphaFold 3 (https://alphafoldserver.com/). Docking simulations were conducted with CB-Dock2 (https://cadd.labshare.cn/cb-dock2/), and resulting complexes were visualized using Discovery Studio and PyMOL.

### Statistical analysis

Data were analyzed using GraphPad Prism 8. Pairwise comparisons were conducted using Student’s t-test, while multi-group comparisons employed one-way or two-way ANOVA. Significance was set at *P* < 0.05, with significance levels denoted as *P < 0.05, **P < 0.01, and ***P < 0.001. Non-significant differences are marked as “ns.”

## Data Availability

All data are available in the figures or can be provided upon request.

## SUPPLEMENTAL MATERIAL

Table S1 jvi.02121-25-s0001.docx Primer sequences used in this study.

ASM does not own the copyrights to Supplemental Material that may be linked to, or accessed through, an article. The authors have granted ASM a non-exclusive, world-wide license to publish the Supplemental Material files. Please contact the corresponding author directly for reuse.
