# Supplementary material for: Small-molecule PS10 inhibits PRRSV replication by targeting HSP90 and multiple viral non-structural proteins
Source: J Virol. 2026 Feb 18;100(3):e02121-25. doi: 10.1128/jvi.02121-25 (PMC13011348; doi:10.1128/jvi.02121-25)
Supplement: Table S1 — Primer sequences used in this study. [file jvi.02121-25-s0001.docx]

**Table S1. Primer sequences used in this study.**

| Primer name^a^ | Sequence (5’-3’) |
| --- | --- |
| PRRSV-N-F | AAAACCAGTCCAGAGGCAAG |
| PRRSV-N-R | CGGATCAGACGCACAGTATG |
| gRNA-F | CTCCACCCCTTTAACCATGTC |
| gRNA-R | AATGCACGTGGCAACGTCCAC |
| sgRNA2-F | CTCTCCACCCCTTKAACCAACTTT |
| sgRNA2-R | CGGAGCAAACCAGTCTGATGC |
| sgRNA3-F | CTCCACCCCTKTAACCATAGTG |
| sgRNA3-R | CCCCTAACCAGCGGAAACCA |
| sgRNA4-F | CTCCACCCCTTTMACCTGGAA |
| sgRNA4-R | TGAGGACTTTTGCGAATCGTCG |
| sgRNA5-F | CTCCACCTTTARCCTGTCT |
| sgRNA5-R | CCAATCTGTGCCATTCAGCTC |
| sgRNA6-F | CTCCACCCCTTTAACCAGAGTTT |
| sgRNA6-R | CTCCACCCCTTTAACCAGAGTTT |
| sgRNA7-F | CTCCACCCCTWTAACCACGCAT |
| sgRNA7-R | ACCCAGCATTTGGCACAGCT |
| mGAPDH-F | TGACAACAGCCTCAAGATCG |
| mGAPDH-R | GTCTTCTGGGTGGCAGTGAT |
| pHPRT1-F | TGGAAAGAATGTCTTGATTGTTGAAG |
| pHPRT1-R  mHSP90AA1-F  mHSP90AA1-R  mHSP90AB1-F  mHSP90AB1-R  mIL-6-F  mIL-6-R  mIL-8-F  mIL-8-R  mTNF-α-F  mTNF-α-R | ATCTTTGGATTATGCTGCTTGACC  AGATAAACCCTGACCATTC  TTCATCAATACCCAGACC  ATCACCCAAGAGGAGTATGG  CGACGAGGAATGAATAGCA  AGAGGCACTGGCAGAAAAC  TGCAGGAACTGGATCAGGAC  CTGGCGGTGGCTCTCTTG  CCTTGGCAAAACTGCACCTT  TCTGTCTGCTGCACTTTGGAGTGA  TTGAGGGTTTGCTACAACATGGGC |
| hIL-6-F | GTAGTGAGGAACAAGCCAGAG |
| hIL-6-R | GCATTTGTGGTTGGGTCA |
| hIL-8-F | GCTAAAGAACTTAGATGTCAGTGC |
| hIL-8-R | CTCAGCCCTCTTCAAAAACTTCTC |
| hTNF-α-F | CCGAGTGACAAGCCTGTAG |
| hTNF-α-R | GGTCTGGTAGGAGACGGCG |
| hGAPDH-F | AAGGTCGGAGTCAACGG |
| hGAPDH-R | GGAAGATGGTGATGGGATT |

^a^ The prefix letters in the primer names indicate the species of origin: "m" for monkey, "p" for pig, and "h" for human.
